# Evaluation of the Effectiveness of Comprehensive Smoke-Free Legislation in Indoor Public Places in Shanghai, China

**DOI:** 10.3390/ijerph16204019

**Published:** 2019-10-21

**Authors:** Yanxia Wei, Ron Borland, Pinpin Zheng, Hua Fu, Fan Wang, Jingyi He, Yitian Feng

**Affiliations:** 1 Department of Preventive Medicine and Health Education, School of Public Health, Fudan University, Shanghai 200032, China; 17111020015@fudan.edu.cn (Y.W.); hfu@fudan.edu.cn (H.F.); 16211020052@fudan.edu.cn (J.H.); 15211020019@fudan.edu.cn (Y.F.); 2 Melbourne School of Psychological Sciences, The University of Melbourne, Melbourne 3010, Australia; rborland@unimelb.edu.au; 3 Department of Politics, East China Normal University, Shanghai 200241, China; wangfan512@126.com

**Keywords:** comprehensive smoke-free law, SHS, PM2.5, indoor public places, compliance

## Abstract

This study evaluated compliance with the comprehensive smoke-free law in public indoor places introduced in Shanghai in March 2017. Observations and PM2.5 monitoring over 30 min intervals in 8 types of the venue were conducted three times: within a month before implementation and 3- and 12-months post implementation. Observations of evidence of smoking decreased from 66.2% before legislation to 52.8% three months after (*p* = 0.002) and 49.7% one year after (*p* < 0.001). The density of lit cigarettes also reduced significantly after implementation (*p* < 0.001). When adjusting for outdoor, indoor PM2.5 levels were significantly lower after the legislation, but only by a small amount (three months later: −0.27, *p* = 0.08; one year later: −0.12; *p* = 0.03). Evidence of compliance was weakest in farmer’s markets and bars, and smoking in male toilets did not change significantly. The reduction in smoking was affected by the management performance of their obligations. The comprehensive smoke-free law led to modest reductions in smoking and PM2.5 levels as a result, but from levels suggesting quite high levels of pre-compliance. However, compliance was limited in some areas, suggesting more effort is required on management to gain better compliance in some places like farmer’s markets, bars, and toilets.

## 1. Introduction

Secondhand smoke (SHS) remains a major public health issue in the world [1]. SHS causes more than 890,000 premature deaths globally every year [2]. Exposure to SHS can lead to cardiovascular and respiratory diseases and some adverse reproductive outcomes such as infertility, low birth weight, infant death and so on [3,4]. China is the largest producer and consumer of tobacco in the world [5] with more than half (52.1%) of males smoking regularly [6]. Smoking in indoor public places is also very common. In China, 72.4% of nonsmokers reported being exposed to SHS and more than 100,000 people die from it annually [7]. According to the 2015 national adult tobacco survey: 54.3% of respondents saw someone smoking at the workplace, 76.3% reported having seen smoking in restaurants, and even more attendees reported smoking in bars, 93.1% [8].

The only intervention shown to fully protect people from the health dangers of SHS is establishing environments that are completely smoke-free [9,10]. On 29 March 2004, the Republic of Ireland became the first country in the world to implement 100% smoke-free legislation [11], and as at 2016 nationally comprehensive smoke-free legislation was in place for almost 1.5 billion people in 55 countries [12]. However, there are still many countries that have not realized smoke-free.

China ratified the WHO Framework Convention on Tobacco Control (FCTC) in 2005 [13], Article 8 of the FCTC calls for the adoption of the effective legislative measures to achieve a 100% smoke-free compliance in all indoor public places, workplaces and public transport [14], which have not been met yet. Between 2009 and 2013, nearly half of all provincial capitals had passed tobacco control legislation [13], but all of them did not realize 100% smoke-free. In 2015, Peking enacted the most restricted tobacco control law in China and realize 100% smoke-free, then other cities started to follow it. 

On 1st March 2017, Shanghai implemented a comprehensive law following Peking. Article 6 stipulates that smoking is prohibited in indoor public places, indoor workplaces, and public transportation, and article 9 require managers of the above smoke-free venues to bear the obligation to post no-smoking signs, remove ashtray and ashtray equivalents and so on. We believed that in China, with the largest smokers and populations in the world, national smoke-free legislation will make a great contribution to tobacco control in the world. However, until now, there are only a few pieces of evidence to demonstrate the impact of the smoke-free policy, which is essential to advocate for national law. We searched the related articles in PubMed in July 2019, there were only three articles that evaluated the 100% smoke-free law in China. Two of them from the same population survey, which showed the reported SHS exposure rates in public places declined [15] and the smoking prevalence in adults also decreased [16] in Beijing after the one year implementation. In the other article also from Beijing, less smoking was observed (from 40.3% to 14.8%) and posting of no-smoking signage increased (from 52.6% to 82.4%) in restaurants after the law came into effect [17]. These evidences are too weak to understand law enforcement in China because there are only a few simple indexes in these studies. 

Our study assessed compliance with the Shanghai law using multiple methods, direct observation, monitoring of air quality and observation of efforts to enhance compliance, to know more details of law enforcement and provide evidence for national law. We hope this study can provide sound evidence to promote a smoke-free China and provide a reference for policymaking in tobacco control in other countries.

## 2. Materials and Methods

### 2.1. Procedure

Observations and PM2.5 monitoring were conducted within a month before implementation (February 2017) and 3 months post implementation in May 2017 and again 12 months post implementation. The same methods were used in all three observation periods. 

Indoor public places were included if they met the following two criteria: smoking was allowed before new law; entry was allowed. Multistage sampling was adopted to recruit venues. Eight types of venues were targeted: office buildings, hotels (four-star hotel and a few low-level hotels), restaurants (Chinese restaurant), bars, Karaoke (KTV), gaming rooms, farmer’s markets, buses and railway stations. First-stage: all eight urban regions (Huangpu, Pudong, Xuhui, Changning, Jing’an, Putuo, Hongkou, and Yangpu) and four of eight suburbs (Minhang, Baoshan, Songjiang, Jiading) in Shanghai were selected, except for bus and railway stations, which are limited in number, so we chose the eight main stations. We chose 20 venues from each of the other 7 venue types (2 each from the urban regions and one from each suburb). We chose staircases as the inspection point in office buildings because we could not enter office areas. Moreover, according to our previous observation, smoking in office buildings is also likely to occur at staircases. For KTVs and hotels, patrons mainly stay in their private room. Considering this limitation, we chose the corridor of KTVs and the lobby in hotels as the inspection point. For other venues, the inspection point is the main business area. Due to the high smoking prevalence among males, where possible, we also made observations in the male toilets. 

The three trained investigators formed a group and surveyed in one district each day during the busy hours of each venue. To ensure validity, investigators entered each venue as guests, investigated for 30 min. One observed and filled the questionnaire, and two investigators were responsible for the PM2.5 concentration detection at the corresponding indoor inspection points. Survey time ranged from 8 am at the farmer’s markets to 11 pm in bars, depending on the peak business hours of different venues. Office buildings were only observed on weekdays. We followed the same schedule in the three-wave surveys. 

### 2.2. Data Collection

Evidence of smoking is a binary variable based on observations of any observed smoking, presence of cigarette butts and the smell of smoke. The number of lit cigarettes was recorded using the average of four observation points (0 min, 10 min, 20 min, 30 min) within 30 min. We also recorded the number of patrons at each point and reported mean levels. The density of lit cigarettes equals the number of lit cigarettes divided by the number of patrons multiplied by 100 to give percentages. Observations of smoking were also made in the male toilets. The venue manager’s performance of compliance obligations was assessed as a binary variable: doing all of posting no-smoking signs, not having a smoking section and no ashtrays and ashtray equivalents present versus all others. 

PM2.5 levels were recorded using a TSI SidePak Model AM510 Personal Aerosol Monitor) fitted with a 2.5 μm impactor. The airflow rate was set at 1.7 L/min, and the logging interval was 1 min. A calibration factor of 0.295 was applied to all raw measurements to correct for the properties of SHS particles [18]. The monitor was calibrated to zero each time before use. The investigators carried the monitor in a small bag with a short length of tubing attached to the inlet and left protruding to the outside. When entering an indoor public place, the investigator sat in the central area or stood in the corridor and staircase and detected PM2.5 level for about 30 min. The PM2.5 concentration in toilets was also measured for 30 min. The monitor was placed as close as possible to the breathing zone, away from any ventilation opening and not within 1 m of any smoking people. Outdoor PM2.5 measurement was carried out for 5 min before entering each venue. The outdoor inspection point was located away from any obvious concentrated source of PM2.5 as practically possible (e.g., venting outlets from kitchens) or sources of fire.

### 2.3. Statistical Analysis

The numbers of people in venues and the density of lit cigarettes were not normally distributed, so mean, median, and interquartile range is reported. We chose not to record the number of people and cigarettes in railway and bus stations because there were too many people to count, therefore all the analysis concluding the number of people and cigarettes did not include the railway and bus stations. The comparison of dichotomous items of different times using Generalized Linear Mixed Model (GLMM) with each place as random effects. The package is “mlmRev” in R. Binomial family with logit link were used. Continuous variables compared using nonparametric test for related samples in SPSS for they do not meet the requirement of GLMM. 

Time-weighted average PM2.5 concentrations were calculated for each visit. Because the PM2.5 concentrations were log normally distributed geometric means (GM) and 95% CI are reported. We compared values between indoor and outdoor, indoor and toilet using Paired Sampled T-Tests. Three times of PM2.5 value was compared using the “nlme” package in R. Gaussian family with logit link were used. All analyses were conducted in SPSS 25.0 (SPSS, IBM, Armonk, NY USA) and R 3.5.2 (R Foundation for Statistical Computing, Vienna, Austria) (Manufacture, city, state abbrev., country). 

## 3. Results

### 3.1. Sample Characteristics

Sampling details are shown in Table 1. In the first survey wave, data from 1 bar, 1 restaurant were lost because of a machine fault, and 1 farmer’s market and 1 office building’s toilet could not be located, so we did not try to resurvey them. Besides, we choose not to survey male toilets in gaming rooms, hotels, and farmer’s markets, because most of these venues did not have their toilet. In total there were 145 indoor public places with 85 male toilets that were monitored three times. There was a small amount of missing data, and none of them exceeded 20%.

### 3.2. Comparation of Observational Items before and after Legislation

Notably, the level of observed smoking-related objects was limited before the ban, suggesting some degree of either enforcement or voluntary changes in behavior in anticipation (see Table 2). Compared with before the legislation, tobacco smell, cigarette butts, lit cigarettes and total evidence of smoking all decreased significantly at three months and stayed at the lower level one year later. In all, evidence of smoking significantly decreased from 66.2% (before legislation) to 52.8% (three months later) to 49.7% (one year later), the latter small change not being significant. Similarly, the fewer density of cigarettes was observed after legislation. In venues where smoking occurred at all three times, the density of cigarettes observed also declined (N = 28, *p* 1 < 0.001, *p* 2 = 0.005). The number of total people in venues was similar (*p* > 0.05) across the three survey periods. 

As for venue management’s compliance with the law, there were significantly more no-smoking signs and less ashtray and ashtray equivalents after legislation and the presence of smoking section did not change significantly. There were already a high proportion of venues that posted no-smoking signs, had no smoking sections and ashtrays before legislation. The number of venues that performed all of the above three obligations raised significantly after law.

For all of the above items except for lit cigarettes, the differences between three months later and one year later were not significant. Smoking in male toilets did not significantly change after legislation and in nearly half (47.4%) of the venues, someone was observed smoking in the toilet one year after the new law was introduced. Smoking at doorways did not change significantly three months later, but it became significantly higher one year after legislation (Table 2). 

As indicated in Table 3, in venues where management complied better with the law (i.e., moved from failure to meeting obligations), evidence of smoking and the density of lit cigarettes both decreased significantly at both 3 months and one year later. Where obligations were met on both occasions, evidence of smoking dropped significantly at 3 months (but not significantly at one year), while the density of lit cigarettes reduced both after 3 months and one year. Similarly, where obligations were not met at either time the density of lit cigarettes became significant less one year later, even though overall compliance did not change. In the small number of venues where performance of obligations declined there were no significant changes in either outcome. Not surprisingly, overall the level of evidence of smoking and the density of lit cigarettes in venues was lower where management complied with their obligations compared with those not fully compliant.

### 3.3. Change of Evidence of Smoking and Density of Cigarettes after Legislation in Different Venues and Districts.

Table 4 presents data on trends by individual venues. Due to the small samples, there is very limited power especially for the assessment of signs of smoking. That said, one-year levels were lower in all seven venues, which had not been subject to prior total bans, but increased marginally (non-significantly) in stations where it was already prohibited. Similarly, there were numerical declines in the density of lit cigarettes in all seven venues, and this was significant in two cases at one year (farmers markets and office buildings) and clear trends in others. It is notable that at one year, no lit cigarettes were observed in more than half of all venues. There was most serious evidence of smoking in the following venues both before and after legislation: farmer’s markets, office buildings, and bars. The median of density of lit cigarettes in farmer’s markets and bars were highest compared to all the other places, although they all had significantly and near significant dropped compared with before legislation. After legislation, evidence of smoking and the density of lit cigarettes both dropped significantly in both urban and suburban venues. Levels were comparable between these two districts types one year after legislation as seen in Table 4.

### 3.4. PM2.5 Concentrations

**Figure 1:**
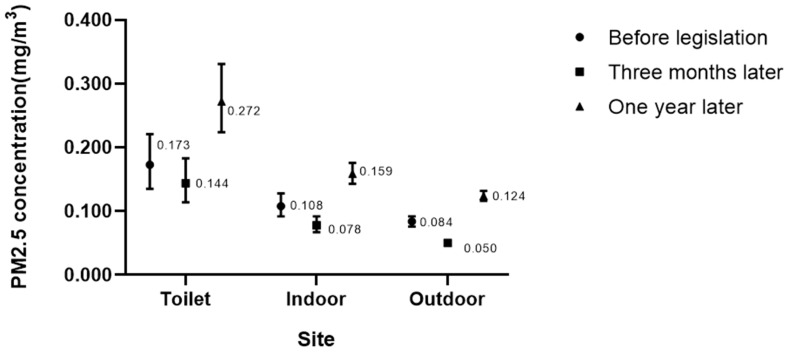
Description of PM2.5 value.

PM2.5 values in male toilets were significantly higher than in the main indoor areas, and the indoor value was significantly higher than outdoors in all three surveys. There was a significant difference among the outdoor values in three waves (*p* < 0.001), with the lowest level detected before the legislation and the highest detected one year after the legislation (Figure 1). Generalized mixed model analysis indicated that outdoor PM2.5 concentrations significantly influenced the indoor value (positive relationship as would be expected), but had no significant effect on the toilet levels. Levels were slightly lower overall after the ban suggesting a small effect on PM2.5 level in the venue. When adjusting for outdoor values, indoor PM2.5 value three months later and one year later all significantly decreased from before legislation, but compared with concentrations after three months, PM2.5 values after one year increased significantly (B = 0.15, *p* = 0.011) even though still significantly lower than the baseline. All these differences were quite small. However, PM2.5 values recorded in toilets were significantly increased one year after legislation. (Table 5)

## 4. Discussion

One year after implementation, Shanghai’s comprehensive smoke-free law has achieved significant reductions in smoking and parallel improvements in indoor air quality. However, the latter effects are small, and only apparent when controlling for outdoor air quality. Evidence of smoking dropped from 66.2% before legislation to 52.8% after three months and to 49.7% after one year, and totally reduced by 25%. The density of cigarettes in the venue also decreased, even in some venues where smoking occurred and in all three survey periods this decrease remained significant. Notably, PM2.5 levels in all indoor public places slightly but significantly dropped after the law was implemented.

The venue managers showed good compliance with the legislation. Less than one month before the new law, there was already a high proportion of venues posting no-smoking signs (73.8%), having no smoking section places (95.8%) and ashtray and its equivalents (66.9%). It is reported that in three main railway stations [19] and some hospitality places [20], smoking sections were abolished just before the legislation. After legislation, more venues posted no-smoking signs and canceled the provision of ashtrays and its equivalents. When we combined all three items, the total performance of obligation also increased. It is clear from the data, that smokers’ behavior is affected by management, with smoker’s compliance higher when managers perform their obligations better when controlled for legislation. Even in venues where the compliance of management didn’t improve after the new law, smoking in indoor places significantly improved, which suggests that the legislation itself can change smoker’s behavior to some extent independent of management behavior. Further, our results demonstrate that the flow of people did not reduce after legislation, which is the same as other similar researches [21,22,23]. Some studies even found smoke-free environments had positive impacts on venue profit and value [24,25], which should encourage operators to enhance enforcement. 

Smoking in male toilets did not change at all, with smoking detected in almost half of the venues (47.4%) one-year post ban. Toilets are places that can be easily ignored by enforcers and management. Another interesting phenomenon is the increasing number of observed smokers smoking in doorways when smoking was prohibited in the venue. Surada et al. found SHS in main entrances (outdoors) were very similar to those in adjacent indoors and SHS from outdoors settings drifts to indoors [26]. Laws need to include prohibitions on smoking in doorways as patrons need to enter or leave through them, and there is a risk of the smoke contaminating the indoor areas. In this study smoking and the density of cigarettes decreased three months later and one year after the ban was formally implemented, but overall there was no change between three months and one year later. In contrast, Fong’s study showed that smoke-free policies in France lead to the near total elimination of smoking after one year and continuously reduced five years later [27]. Other studies also certified the constant and stronger effect of comprehensive legislation [28,29]. There is strong evidence that 100% smoke-free law had a sustained effect and can totally stop observed smoking. Shanghai’s comprehensive smoke-free law may be less successful compared with other countries at this point, so more efforts should be put into law enforcement. 

There was some variation in the extent of compliance with obligations and compliance by smokers across venues with both poor in some venues especially farmer’s markets and bars, but even here, the density of smoking declined. This supports the view that Shanghai’s legislation is partially successful with occasional violations, common in some places, but less smoking overall. The legislation has been more successful in reducing the number of people smoking rather than creating environments where there is no smoking. One year after the new law, over half of the farmer’s markets (88.9%), office buildings (70.09%), and bars (63.16%) had evidence of smoking. Bars and farmer’s markets had both the highest evidence of smoking and the density of cigarettes. International studies have shown that there are more smoking in bars than in other places [30,31,32], which is consistence with the findings in our study as the highest PM2.5 levels and number of cigarettes occurred in bars. However, almost no study has focused on smoking in farmer’s markets where we also found high levels of non-compliance demonstrating that such places should be a focus for improving compliance. If compliance is to be improved it is likely to involve further public education about the importance of protecting non-smokers from passive smoking, and efforts targeting management of areas where compliance with their obligations was particularly poor. 

As the results show, the outdoor PM2.5 values were much higher than most of the other similar research, and the three-time waves of outdoor levels were significantly different, so the indoor PM2.5 concentrations did not decrease directly as other researches [33,34]. When adjusting outdoor background value, the PM2.5 level was slightly dropped three months later then slightly increased one year later but still lower than before legislation. The SHS in indoor public places declined following the reduction of smoking. Protano et al. also found the PM concentrations closely related with smoking in indoor places [35]. Male toilets had higher PM2.5 concentrates than indoor and its PM2.5 levels slightly but significantly increased one year later. The less but significant change of PM2.5 concentration is relative to our very low baseline number of cigarettes (0.25 (0.00~1.50)). The legislation had some effect on SHS in the venue, but it did not remove it completely and the SHS in male toilets even increased. When combined with our observational outcome that smokers in male toilets did not drop after the new laws were introduced, it is clear that smoking in toilets needs further attention by compliance enforcement. 

One limitation of our study is that our pre-ban survey was too close in time to when the law came into force. A lot of changes appear to have happened, presumably because of advocacy, and/or preparation for the formal implementation, so what we measured at baseline almost certainly reflects a higher level of control over indoor smoking than we would have observed before the law was announced. This means that the effects we measured may be somewhat conservative and the actual improvements are likely to be greater. Secondly, we only chose the lobby of hotels and the corridor of KTVs because of fund limitations. Some studies have found that there are serious SHS in guest rooms of hotels and KTVs [36,37], so our findings may not generalize areas where we did not survey. Thirdly, PM2.5 value is not a sensitive index for SHS when there is a small number of cigarettes and when the outside level is relatively high, as it was in our study. Although we adjusted the outdoor value and found an effect, there is some doubt over the magnitude and thus the likely benefits. That said, the levels we detected are consistent with indoor exposure typically being quite low post-ban, and thus of limited concern. Fourthly, This study adopted the before-and-after studies with no control area for reference, which were unable to control for possible confounders and changes in secular trends over time [38]. These unsolvable changes may be small within one year as we did not observe obvious changes from venues. But for much longer investigation periods, find another area for control is better.

## 5. Conclusions

The comprehensive smoke-free law can effectively reduce smoking in indoor public places and protect nonsmokers from SHS. Our study detailed the areas that need to be enhanced in law enforcement and the new challenges that arise after legislation is introduced, which are the potential problems that many other countries in the world may encounter in the enforcement of tobacco control legislation since it is difficult to totally eliminate smoking in indoor public places even in countries with national comprehensive law, like France [27], Italy [33], Ireland [39], Uruguay [40] and so on. Importantly, we also found that management participation is an important factor in the elimination of smoking in public places and this needs to be the target of compliance enforcement.

## Figures and Tables

**Table 1:** Sample details.

Site	Inspection Point	Number	Time
Office building	Staircase/male toilet	20/20	10:00–11:30
Restaurant	Main hall/male toilet	19/19	11:30–14:00 17:00–19:00
Bar	Main hall/male toilet	19/19	21:00–23:00
Gaming room	Main hall/male toilet	20/0	15:30–17:00
Hotel	Lobby/male toilet	20/0	14:00–15:30
KTV	Corridor/male toilet	20/20	19:00–21:00
Farmer’s market	Main hall/male toilet	19/0	8:00–10:00
Bus and railway station	Waiting hall/male toilet	8/8	10:00–17:00

**Table 2:** The comparation of observational items before and after legislation.

Observational Items.		Time			*p* 1	*p* 2
		Before Legislation	Three Months Later	One Year Later		
Evidence of smoking (% (*N*))	Tobacco smell	53.8 (78)	29.2 (42)	29.7 (43)	<0.001	<0.001
	Cigarette butts	43.7 (62)	29.7 (43)	26.6 (38)	0.003	<0.001
	Lit cigarettes	52.4 (76)	40.7 (59)	31.0 (45) *	0.020	<0.001
	Any	66.2 (94)	52.8 (76)	49.7 (71)	0.002	<0.001
Density of lit cigarettes (mean/median (IR)) *N* = 122		5.99/1.23	2.71/0.00	1.50/0.00	0.005	<0.001
		(0.00~8.37)	(0.00~2.49)	(0.00~0. 93)		
Number of people (mean/median (IR)) *N* = 127		34.35/18.00	32.48/19.00	30.32/17.25	0.474	0.115
		(7.00~43.75)	(7.25~32.00)	(6.25~37.0)		
Performance of obligation (% (*N*))	Posting no-smoking signs	73.8 (107)	88.3 (128)	91.0 (132)	<0.001	<0.001
	Have no smoking section	95.8 (138)	98.6 (142)	98.6 (143)	0.126	0.124
	Have no ashtray and ashtray equivalents	66.9 (95)	84.1 (116)	89.0 (129)	<0.001	<0.001
	All	51.1 (72)	73.6(103)	80.0(116)	<0.001	<0.001
Smoking in toilet (% (*N*))		48.2 (40)	35.4 (29)	46.3 (38)	0.075	0.800
Smoking at doorway (% (*N*))		21.5 (31)	29.6 (42)	39.3 (57) *	0.096	<0.001

Note: *p* 1 is the outcome of comparation between before legislation and three months later, *p* 2 is between before legislation and one year later. Significant codes indicate between three months later and one year later: ‘**’ < 0.01, ‘*’ < 0.05.

**Table 3:** Evidence of smoking and density of lit cigarettes following the change of venue manager’s performance of obligation.

	Evidence of Smoking (%)				Density of Lit Cigarettes (Mean/Median (IR))			
Perform of Obligation	Both Comply all	Neither Comply all	Increase from Baseline	Decrease from Baseline	Both Comply all	Neither Comply all	Increase from Baseline	Decrease from Baseline
Before legislation	59.6	85.2	70.0	40.0	3.84/0.00	14.50/10.73	6.91/1.27	1.32/0.00
					(0.00~2.94)	(3.95~18.75)	(0.00~9.50)	(0.00~2.17)
Three months later	42.1	81.5	45.0	60.0	0.67/0.00	7.75/7.41	2.34/0.00	1.82/0.00
					(0.00~0.77)	(1.59~14.29)	(0.00~0.92)	(0.00~2.77)
*p* 1	0.031	1.000	0.013	0.500	0.002	0.136	0.003	0.593
(*N*)	(*N* = 57)	(*N* = 27)	(*N* = 40)	(*N* = 10)	(*N* = 50)	(*N* = 23)	(*N* = 38)	(*N* = 9)
Before legislation	55.7	89.5	70.8	55.6	1.54/0.00	17.44/13.03	7.01/3.85	2.79/0.00
					(0.00~2.61)	(6.41~24.11)	(0.00~9.86)	(0.00~6.25)
One year later	47.5	78.9	43.8	55.6	0.80/0.00	3.83/1.03	1.44/0.00	1.37/0.00
					(0.00~0.94)	(0.00~8.40)	(0.00~0.00)	(0.00~1.36)
*p* 2	0.332	0.500	0.004	1.000	0.031	0.001	<0.001	0.225
(*N*)	(*N* = 61)	(*N* = 19)	(*N* = 48)	(*N* = 9)	(*N* = 53)	(*N* = 18)	(*N* = 47)	(*N* = 9)

Note: *p* 1 is the outcome of comparation between before legislation and three months later, *p* 2 is between before legislation and one year later.

**Table 4:** Changes of evidence of smoking and density of lit cigarettes in different venues and districts.

Venue		No Lit Cigarettes in all of Three Times (% (*N*))	Before Legislation	Three Months Later	One Year Later	*p* 1	*p* 2
Farmer’s market	Evidence of smoking (% (*N*))		94.4 (17)	94.7 (18)	88.9 (16)	0.969	0.554
	Density of lit cigarettes (mean/median (IR)) *N* = 18	0.0 (0)	3.02/2.69 (1.28~4.03)	1.12/0.71 (0.51~1.45)	1.55/1.31 (0.00~2.55)	0.002	0.008
Hotel	Evidence of smoking (% (*N*))		40.0 (8)	21.1 (4)	10.0 (2)	0.149	0.065
	Density of lit cigarettes (mean/median (IR)) *N* = 18	66.7 (12)	1.47/0.00 (0.00~0.96)	1.19/0.00 (0.00~0.00)	0.04/0.00 (0.00~0.00)	0.934	0.560
Bar	Evidence of smoking (% (*N*))		83.3 (15)	78.9 (15)	63.2 (12)	0.802	0.163
	Density of lit cigarettes (mean/median (IR)) *N* = 16	0.6(1)	17.46/14.53 (4.79~23.52)	4.79/3.24 (0.00~8.10)	5.44/0.44 (0.00~11.77)	0.077	0.052
Office building	Evidence of smoking (% (*N*))		90.0(18)	50.0(10)	70.0(14)	0.014	0.116
	Density of lit cigarettes (mean/median (IR)) *N* = 16	43.8(7)	8.14/2.58 (0.00~8.82)	2.19/0.00 (0.00~3.14)	0.62/0.00 (0.00~0.00)	0.289	0.034
Restaurant	Evidence of smoking (% (*N*))		52.6 (10)	21.1 (4)	27.8 (5)	0.035	0.099
	Density of lit cigarettes (mean/median (IR)) *N* = 17	58.9(10)	3.54/0.00 (0.00~5.15)	0.25/0.00 (0.00~0.00)	0.64/0.00 (0.00~0.00)	0.123	0.198
KTV	Evidence of smoking (% (*N*))		60.0 (12)	60.0 (12)	55.0 (11)	1.000	0.699
	Density of lit cigarettes (mean/median (IR)) *N* = 17	47.1(8)	1.81/0.00 (0.00~1.92)	5.34/0.00 (0.00~6.20)	0.28/0.00 (0.00~0.33)	0.391	0.932
Gaming room	Evidence of smoking (% (*N*))		65.0 (13)	55.0 (11)	36.8 (7)	0.273	0.055
	Density of lit cigarettes (mean/median (IR)) *N* = 20	35.0 (7)	7.43/7.42 (0.00~14.89)	4.10/0.39 (0.00~8.33)	2.07/0.00 (0.00~1.93)	0.385	0.082
Railway and bus station	Evidence of smoking (% (*N*))		14.3 (1)	25.0 (2)	25.0 (2)	0.609	0.609
District							
Urban	Evidence of smoking (% (*N*))		60.5 (69)	51.3 (59)	45.6 (52)	0.061	0.005
	Density of lit cigarettes (mean/median (IR)) *N* = 95	37.9(36)	5.68/1.00 (0.00~0.080)	2.50/0.00 (0.00~2.00)	1.34/0.00 (0.00~1.00)	<0.001	0.007
Suburb	Evidence of smoking (% (*N*))		89.3 (25)	58.6 (17)	62.1 (18)	0.008	0.013
	Density of lit cigarettes (mean/median (IR)) *N* = 27	29.7(8)	7.22/3.00 (0.00~10.00)	3.52/1.00 (0.00~6.00)	2.15/0.00 (0.00~3.00)	0.276	0.048

Note: *p* 1 is the outcome of comparation between before legislation and three months later, *p* 2 is between before legislation and one year later.

**Table 5:** Change of PM2.5 value after adjusting for outdoor background value.

	Indoor PM2.5 Value			Toilet PM2.5 Value		
	B ^1^	S.E. ^2^	*p*-value	B ^1^	S.E. ^2^	*p*-value
Intercept	−2.30	0.14	< 0.001	−1.52	0.22	<0.001
Legislation (reference = before legislation)						
Three months later	−0.27	0.08	< 0.001	0.01	0.18	0.954
One year later	−0.12	0.03	< 0.001	0.45	0.12	<0.001
Outdoor PM2.5 value	5.67	0.74	< 0.001	2.29	1.41	0.107

B ^1^: regression coefficient. S.E. ^2^: standard error.
